# Single-cell transcriptomics reveals heterogeneity and prognostic markers of myeloid precursor cells in acute myeloid leukemia

**DOI:** 10.3389/fimmu.2024.1494106

**Published:** 2024-12-16

**Authors:** Guangfeng He, Lai Jiang, Xuancheng Zhou, Yuheng Gu, Jingyi Tang, Qiang Zhang, Qingwen Hu, Gang Huang, Ziye Zhuang, Xinrui Gao, Ke Xu, Yewei Xiao

**Affiliations:** ^1^ Department of Hematology, Affiliated Hospital of Southwest Medical University, Luzhou, China; ^2^ Department of Clinical Medicine, Southwest Medical University, Luzhou, China; ^3^ Department of Laboratory Medicine, Southwest Medical University, Luzhou, China; ^4^ First Clinical Medical College, Guangdong Medical University, Zhanjiang, China; ^5^ Department of Oncology, Affiliated Hospital of Southwest Medical University, Luzhou, China; ^6^ Department of Oncology, Chongqing General Hospital, Chongqing University, Chongqing, China; ^7^ Department of Physiology, School of Basic Medical Sciences, Southwest Medical University, Luzhou, China

**Keywords:** acute myeloid leukemia, prognostic biomarkers, immune escape, personalized treatment, immunotherapy

## Abstract

**Background:**

Acute myeloid leukemia (AML) is a hematologic tumor with poor prognosis and significant clinical heterogeneity. By integrating transcriptomic data, single-cell RNA sequencing data and independently collected RNA sequencing data this study aims to identify key genes in AML and establish a prognostic assessment model to improve the accuracy of prognostic prediction.

**Materials and methods:**

We analyzed RNA-seq data from AML patients and combined it with single-cell RNA sequencing data to identify genes associated with AML prognosis. Key genes were screened by bioinformatics methods, and a prognostic assessment model was established based on these genes to validate their accuracy.

**Results:**

The study identified eight key genes significantly associated with AML prognosis: SPATS2L, SPINK2, AREG, CLEC11A, HGF, IRF8, ARHGAP5, and CD34. The prognostic model constructed on the basis of these genes effectively differentiated between high-risk and low-risk patients and revealed differences in immune function and metabolic pathways of AML cells.

**Conclusion:**

This study provides a new approach to AML prognostic assessment and reveals the role of key genes in AML. These genes may become new biomarkers and therapeutic targets that can help improve prognostic prediction and personalized treatment of AML.

## 1 Introduction

AML is a highly heterogeneous hematologic malignancy characterized by clonal proliferation of myeloid precursor cells leading to impaired differentiation and accumulation of immature primitive cells in the bone marrow and peripheral blood (1–3). AML accounts for approximately 80% of adult acute leukemia cases and carries a poor prognosis, especially in elderly patients (4, 5). Despite advances in therapeutic strategies, including chemotherapy, hematopoietic stem cell transplantation, and targeted therapies, overall survival in AML remains poor, with a 5-year survival rate of only 25% to 30% (6). An important reason for this poor prognosis is the high degree of heterogeneity in the biological and clinical manifestations of AML, which highlights the importance of searching for reliable prognostic biomarkers in order to predict the patient’s prognosis and develop a personalized treatment plan (7, 8).

Over the past decade, many studies have been devoted to unraveling the molecular features of AML in an attempt to improve AML therapeutic approaches by identifying gene mutations, chromosomal abnormalities, and gene expression profiles that are associated with disease progression and prognosis (9). For example, AML with NPM1 and CEBPA mutations usually has a better prognosis, whereas AML with FLT3 mutations has a worse prognosis. These differences are critical for the choice of treatment strategies. High-throughput sequencing technologies, particularly RNA sequencing (RNA-seq), have revealed a variety of genes and abnormal signaling pathways that are frequently mutated in AML, providing valuable clues for understanding the pathogenesis of the disease. However, despite these advances, a comprehensive understanding of the molecular mechanisms affecting AML prognosis is still lacking, and the search for reliable prognostic biomarkers remains challenging (10, 11).

In this study, we adopted an integrated multi-omics approach to systematically identify and screen for possible prognostic biomarkers using transcriptomic and epigenetic data from public AML datasets. We performed survival analysis, gene expression analysis, and pathway enrichment analysis to mine genes that may be associated with AML prognosis. In addition, to validate our findings, we performed RNA-seq sequencing from bone marrow samples of 10 AML patients and 10 healthy donors to assess the expression levels of the screened candidate genes.

The main goal of this study was to provide a comprehensive prognostic biomarker analysis of AML, providing insight into the molecular mechanisms behind AML progression. By integrating RNA-seq data from public databases and our own patient cohort, we aim to identify gene signatures that are not only associated with clinical prognosis, but also hope to provide potential targets for the development of personalized treatment strategies. Ultimately, our findings are expected to improve risk stratification in AML and provide a basis for future therapeutic development targeting these prognostic genes.

## 2 Materials and methods

### 2.1 Sample source and collection

A total of 20 bone marrow samples were collected for this study, consisting of 10 bone marrow samples from patients with acute myeloid leukemia (AML) (AML group) and 10 bone marrow samples from healthy individuals (control group). All AML patients were diagnosed by bone marrow smear morphology and cytogenetic testing, and individuals in the healthy control group underwent a thorough physical examination to exclude any history of blood disorders and tumors. The samples were collected from 2023 to 2024 at Zhongshan Campus of Southwest Medical University Hospital and stored in liquid nitrogen for further processing. All subjects signed an informed consent form, and the study was approved by the Ethics Committee of the Affiliated Hospital of Southwest Medical University (Ethics Approval No. KY2024070).

### 2.2 RNA extraction and sequencing

Total RNA was extracted from frozen bone marrow samples using TRIzol reagent (Thermo Fisher Scientific, USA) for RNA extraction according to the manufacturer’s instructions. The quality of extracted RNA was assessed by Agilent 2100 Bioanalyzer to ensure that the RNA Integrity Index (RIN) was greater than 7.0, and the RNA concentration was quantified using Qubit 2.0 (Thermo Fisher Scientific) to ensure that it met the requirements for sequencing. The RNA libraries were constructed using the Illumina TruSeq RNA Library Prep Kit (Illumina, USA) and library quality control was performed by Qubit and Bioanalyzer. All samples were bipartite sequenced on the Illumina NovaSeq 6000 platform with a read length of 150 bp and a target sequencing depth of 50M reads per sample.

### 2.3 Public data sources

The single cell sequencing data used in this study were obtained from the dataset GSE116256 in the GEO database (12). This dataset includes 16 patients at the time of AML diagnosis, 19 patients during treatment, and 5 healthy control donors. Considering our primary focus on the disease pathogenesis of AML, we selected three high-quality samples from each of the patients at the time of AML diagnosis and healthy control donors, excluding the interference of drug treatment. The RNA-seq data for AML were obtained from datasets GSE12417 and GSE71014 in the GEO database, containing 405 and 104 samples, respectively (13). The GSE12417 data was used as a training set during the construction of the prognostic model, while the GSE71014 data was used as an external validation set to evaluate the performance of the model.

### 2.4 Single-cell sequencing data processing and cell type identification

After reading single-cell sequencing data from three AML patients and three normal bone marrow samples, we used the Seurat package to perform initial processing of the data, including quality control, dimensionality reduction clustering, and visualization (14). To ensure that subsequent analyses were based on high-quality sequencing data, we performed stringent quality control criteria on the cells, removing those with fewer than 500 or more than 5000 genes measured, as well as those with a proportion of mitochondrial genes greater than 15%, and avoiding the interference of empty droplets, doublets, and senescent cells. After data standardization and normalization, we performed PCA downscaling analysis. Based on the PCA downscaling results, a batch effect correction was performed using the “harmony” package. Next, we selected the top 20 principal components for cluster analysis, and the cluster resolution was set to 0.3, resulting in 10 cell clusters, which were visualized by UMAP. We performed preliminary cell type annotation for each population with the help of common cell marker genes and “FindAllMarkers” function, and finally identified myeloid precursor cells, monocytes, T cells, erythrocytes, NK cells and B cells.

### 2.5 Cellular communication analysis and pseudo-time analysis

In analyzing cell-cell interactions in the AML tumor microenvironment, we used the “CellChat” package for cellular communication network analysis, which simulates and analyzes cell-cell communication patterns by combining gene expression data with information on known signaling pathways, including ligand, receptor, and cofactor interactions. communication patterns (15). To further explore the developmental trajectories of different cell types in the tumor microenvironment and their dynamics during tumor progression, we used the Monocle R package to perform pseudo-temporal analyses of single-cell RNA sequencing data to reveal key transitions during cell development (16–18). We also used the Slingshot package for pseudo-temporal analysis to further explore the developmental trajectories of cells in single-cell RNA sequencing data. Slingshot is a powerful tool that can efficiently handle data with complex branching structures, helping us to gain a deeper understanding of the changes in the cellular state at the single-cell level (19). With these two analyses, we were able to paint a comprehensive picture of cellular developmental pathways and their dynamic behaviors in the AML tumor microenvironment.

### 2.6 Enrichment analysis

To explore the biological characteristics of myeloid precursor cells, we performed GSEA (Gene Set Enrichment Analysis). The gene sets used for enrichment analysis are differentially expressed genes identified by the “FindAllMarkers” function. We performed enrichment analysis of myeloid precursor cells using the KEGG gene set to identify pathways related to their biological functions. In addition, we performed GO and KEGG enrichment analyses on patients in the high-risk and low-risk groups. The set of genes used for enrichment analysis was derived from genes up regulated for expression in the high-risk group. To facilitate the retrieval of gene sets in the GO and KEGG databases, we used the “clusterProfiler” package and visualized the results of the analyses using this package (20). These analyses helped us to gain a deeper understanding of the biology of myeloid precursor cells and patients in different risk groups.

### 2.7 Construction of myeloid precursor cell marker genes and prognostic models

We screened myeloid precursor cells for marker genes using the “FindMarkers” function with a log2FC threshold of 0.25. Then, univariate Cox regression analyses were performed to initially screen out genes with prognostic value from the marker genes. Subsequently, we built multiple prognostic models using the GSE12417 and GSE71014 datasets in combination with 101 algorithm combinations and calculated the average C-index of each model across all cohorts to assess its predictive power. The analysis showed that the model combining the GBM (Gradient Booster) and Lasso (Least Absolute Shrinkage and Selection Operator) algorithms had the highest average C-index and was selected as the final model. The Lasso algorithm was used to identify the most prognostic genes while the GBM algorithm was used to build the final prognostic model, which consisted of 8 genes (21, 22). Finally, we plotted the Kaplan-Meier (K-M) survival curves for each gene as well as the K-M survival curves for the high- and low-risk groups using the “survival” and “survminer” packages to assess the model’s prognostic performance.

### 2.8 Analysis of immune infiltration in high and low risk groups

To gain insight into the relationship between the prognostic impact of myeloid precursor cells and the immune microenvironment, we used the CIBERSORT tool to analyze samples for immune infiltration. Specifically, we performed quantitative assessment of immune cell composition for single gene grouped and risk grouped samples. CIBERSORT utilizes transcriptomic data to make inferences about the relative abundance of immune cell subpopulations, thereby revealing the infiltration status and characterization of immune cells in different risk groups. We also performed immune checkpoint analysis to assess the expression levels of immune checkpoint molecules and their differences in high and low risk groups. The expression of immune checkpoint molecules plays a key role in the suppression and activation of the immune system, and changes in them may affect the immune escape mechanisms of tumors. We analyzed the expression patterns of common immune checkpoints, including PD-1, PD-L1, and CTLA-4, and explored their correlation with high and low risk groups. In addition, we performed an immune function analysis to further reveal the functional status of the immune system in different risk groups by assessing the enrichment of immune-related pathways and functions. This analysis helped us to understand the dynamics of immune function in the tumor microenvironment and its potential impact on disease progression.

### 2.9 Statistical analysis

Statistical analyses were performed using R 4.2.2 64-bit version and its supporting software packages. For continuous variables, the nonparametric Wilcoxon rank sum test was used to assess the relationship between the two groups. The Sperman correlation analysis was used to test the correlation coefficients. All statistical analyses were performed at a level of significance of P<0.05.

## 3 Results

### 3.1 Single-cell data dimensionality reduction clustering and cell type identification

**Figure 1:**
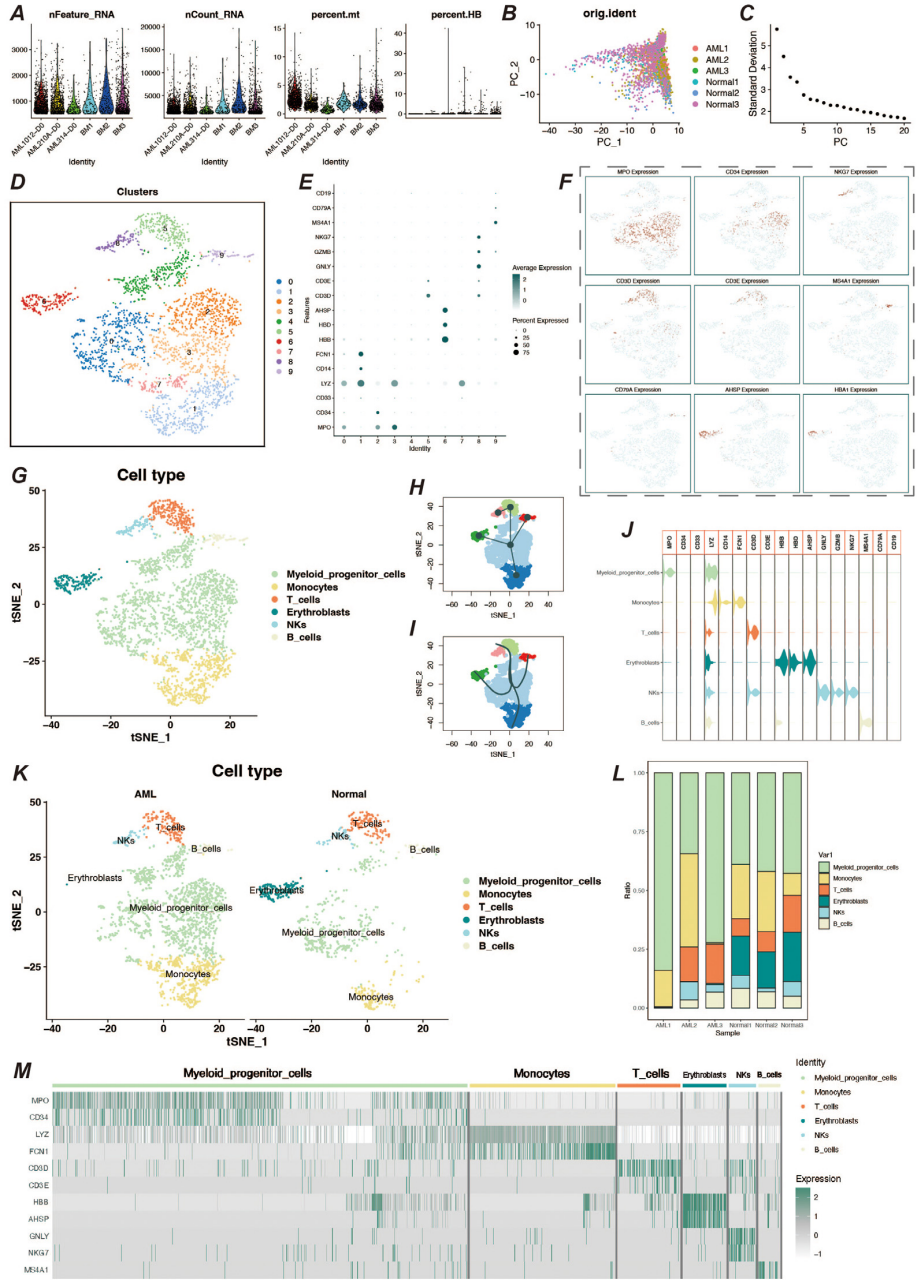
Single-cell data processing and cell type identification. **(A)** Violin plots of sample characteristics after quality control of single-cell data, showing the distribution of gene counts, total counts, mitochondrial ratio, and red blood cell ratio. **(B)** Visualization of cell distribution from different samples in the principal component analysis (PCA) space. **(C)** Elbow plot for dimension selection, used to determine the optimal number of dimensions in the dimensionality reduction process. **(D)** t-SNE dimensionality reduction clustering plot, showing the result of cells being divided into 10 clusters. **(E)** Bubble chart of marker gene expression, displaying the expression levels of marker genes across different cell populations. **(F)** Feature plot of marker gene expression, presenting the distribution of specific marker genes within cell populations. **(G)** t-SNE plot showing the results of cell type identification, displaying the distribution of cell types in two-dimensional space. **(H, I)** Pseudo-time trajectory plots, respectively showing the developmental trajectory of cells in pseudo-time analysis. **(J)** Violin plot of marker gene expression, describing the expression distribution of specific marker genes across different cell populations. **(K)** Cell type identification result display, showing the distribution and quantity of cell types by different groups. **(L)** Bar chart of cell proportions, displaying the proportion of different cell types within the total cell population. **(M)** Heatmap of cell marker gene expression, showing the expression levels of marker genes within various cell populations.

We obtained bone marrow aspiration single cell sequencing data from the GSE116256 dataset for three acute myeloid leukemia patients and three healthy individuals, with samples in the AML group coming from unmedicated patients. We processed the single-cell data using the Seurat package. To remove senescent and low-quality cells, we performed quality control on the cells (**Figure 1A**). After going through a series of steps of normalization, finding highly variable genes, and normalizing the expression matrix, a dimensionality reduction clustering step was performed. After PCA dimensionality reduction, we briefly observed the distribution of sample cells and the contribution of dimensions (**Figures 1B, C**) and selected the top 20 PCs for further dimensionality reduction clustering, and we clustered the cells into a total of 10 cell clusters (**Figure 1D**). We obtained relevant cell marker genes from the CellMarker website (http://xteam.xbio.top/CellMarker/index.jsp) and utilized the expression of these marker genes in the cell clusters for cell type identification (**Figures 1E, F**). Eventually we identified myeloid precursor cells, monocytes, erythrocytes, T cells, NK cells, and B cells (**Figure 1G**). Based on cell types, we observed the general spectrum of cells using the proposed temporal trajectory analysis (**Figures 1H, I**), which verified the accuracy of the myeloid precursor cells we identified. Meanwhile, we also utilized violin plots and heat maps to demonstrate the expression of marker genes in each cell type (**Figures 1J, M**). By comparing the number and distribution of cells in the normal and AML groups, we found that myeloid precursor cells were significantly increased in the AML group (**Figures 1K, L**).

### 3.2 Analysis of cellular communication in the AML tumor microenvironment

**Figure 2:**
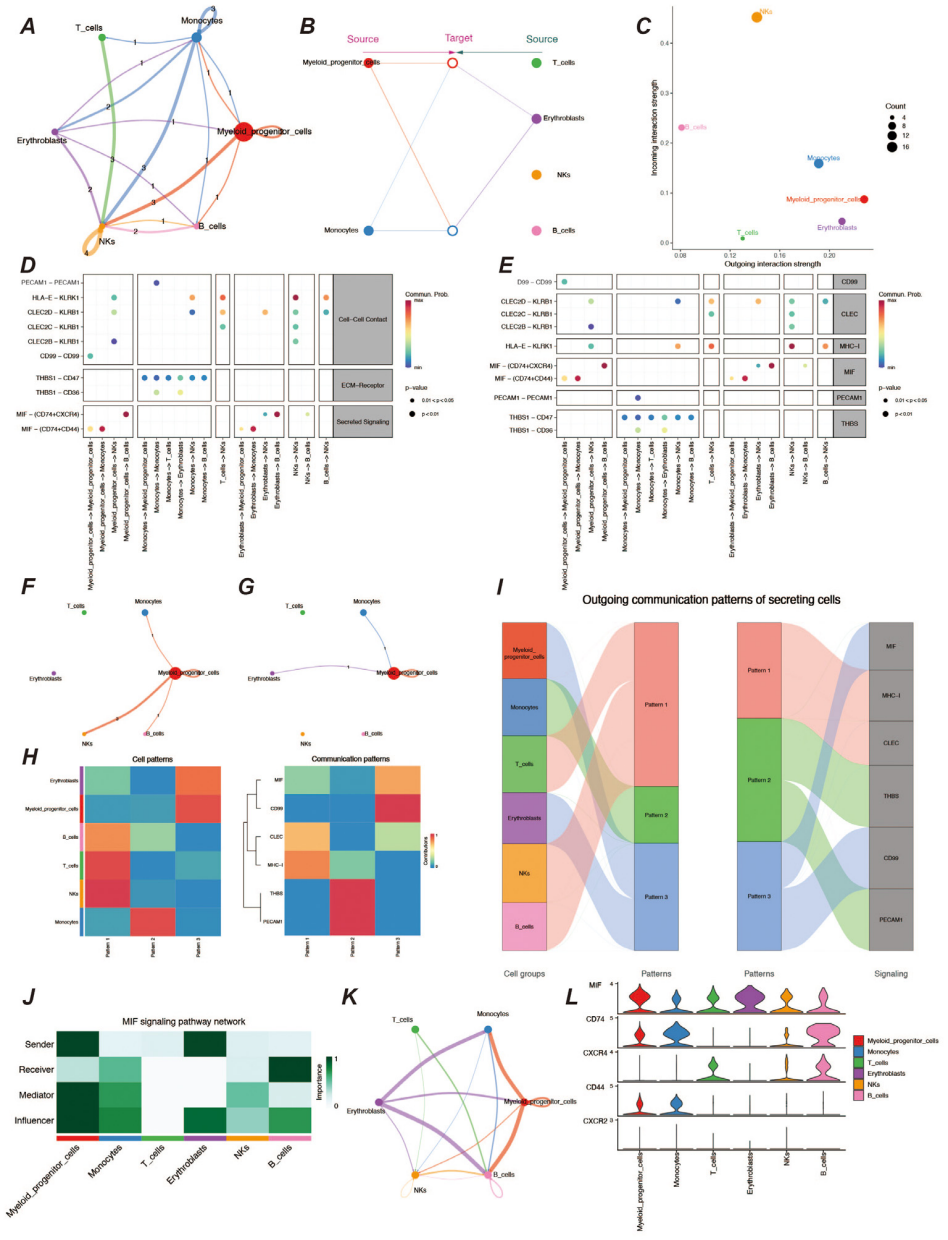
Analysis of cellular communication in the tumor microenvironment. **(A)** Cellular communication chord diagram, where the thickness of the lines represents the frequency of communication between different cells, reflecting the strength of cell interactions. **(B)** Hierarchical diagram of cellular communication, showing the communication relationships and hierarchical structure among cells. **(C)** Scatter plot of signal emission and reception intensity, displaying the distribution of signal emission and reception strength among different cells. **(D, E)** Bubble charts of ligand-receptor communication intensity, respectively showing the communication strength between ligands and receptors. The size and color of the bubbles indicate the strength and frequency of communication. **(F, G)** Chord diagrams of signal emission and reception by myeloid precursor cells, respectively showing the communication patterns of myeloid precursor cells when emitting and receiving signals. **(H)** Heatmap of cellular communication patterns, displaying the communication patterns and intensities between different cell types. **(I)** Sankey diagram of cellular communication patterns, showing the distribution of communication patterns across various cell types. **(J)** Heatmap of role preferences during cellular communication via MIF communication family ligand receptors in different cell types. **(K)** Chordal plot of cellular communication via MIF family ligand receptors. **(L)** Violin plot of MIF family ligand receptor gene expression in various cell types.

To understand the cellular communication between various cell types in the AML tumor microenvironment, we performed cellular communication inference using the CellChat package. Interaction between myeloid precursor cells and NK cells was relatively significant in the tumor microenvironment, and additionally, communication between monocytes in immune cells was also very active (**Figures 2A, F, G**). Because both myeloid precursor cells and monocytes belong to the myeloid lineage, their patterns of autocrine and paracrine communication were similar (**Figure 2B**). However, myeloid precursor cells send signals with higher intensity than monocytes and are the most active presence in the tumor microenvironment. In contrast, myeloid precursor cells received signals at a lower intensity, which may suggest their unregulated presence in the context of AML disease (**Figure 2C**). In addition we observed that the macrophage migration inhibitory factor (MIF) signaling pathway played an important role in the cellular communication process of myeloid precursor cells, which may be related to the large proliferation of myeloid precursor cells (**Figures 2D, E**) (23–25). We also resolved the communication patterns in the tumor microenvironment by NMF analysis, which also helped us to identify the specificity of myeloid precursor cells in communication (**Figures 2H, I**).The MIF pathway played multiple roles in the communication process of myeloid precursor cells, through which myeloid cells mainly communicated with monocytes and B-cells, and the cellular expression of MIF, CD74 and CD44 genes MIF, CD74 and CD44 genes were expressed at high levels in the cells and were the main ligand receptors mediating the communication through this pathway (**Figures 2J–L**).

### 3.3 Pseudo-time analysis and enrichment of myeloid precursor cells

**Figure 3:**
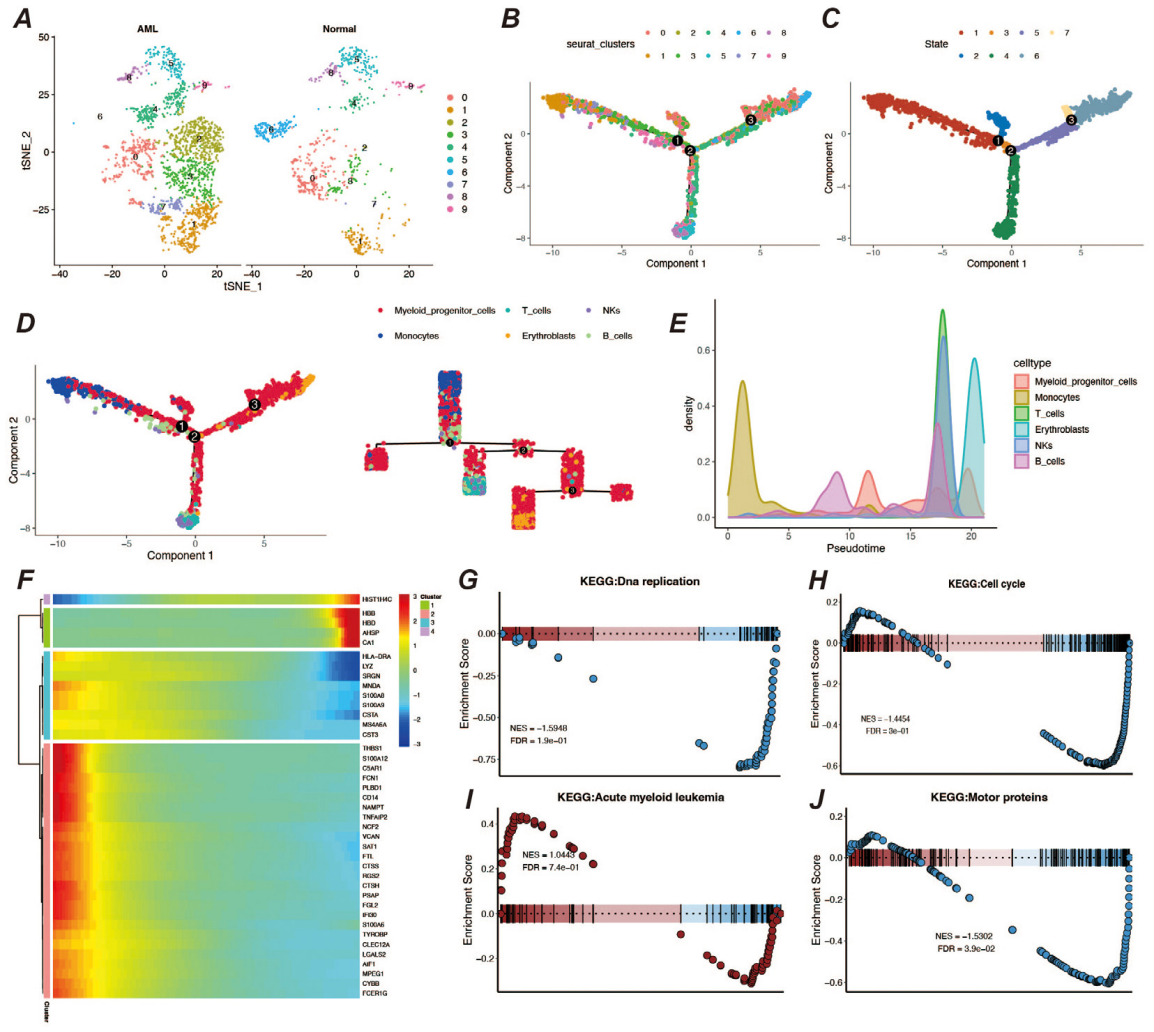
Pseudo-time analysis and enrichment analysis. **(A)** Display of cell clustering results, showing the distribution and quantity of cell types in different groups. **(B, C)** Pseudo-time developmental trajectory plots. **(D)** Distribution of different cell types on the pseudo-time trajectory, with a developmental trajectory dendrogram on the right. **(E)** Cell count peak plot. **(F)** Heatmap of differentially expressed genes in pseudo-time. **(G–J)** Results of GSEA enrichment analysis for myeloid precursor cells.

To further understand the developmental trajectories and lineages in the tumor microenvironment, we performed a proposed-time analysis using the monocle2 package, and the trajectory results presented three distinct nodes and three branches (**Figures 3A–C**). Among them, monocytes, a cell type already present in the tumor microenvironment, underwent extensive proliferative development of myeloid precursor cells on the proposed temporal trajectory, and their peak numbers appeared in the middle and late stages of the proposed time (**Figures 3D, E**). We also observed a number of genes that were significantly differentially expressed at the proposed time, including genes such as AHSP and CA1 that functioned at a late stage, but also genes such as THBS1, CD14, S100A9, and FCN1 that functioned at an early stage of development (**Figure 3F**). By enrichment analysis of myeloid precursor cells, we observed apical enrichment for acute myeloid leukemia (**Figures 3G–J**).

### 3.4 Screening of myeloid precursor cell-related prognostic genes

**Figure 4:**
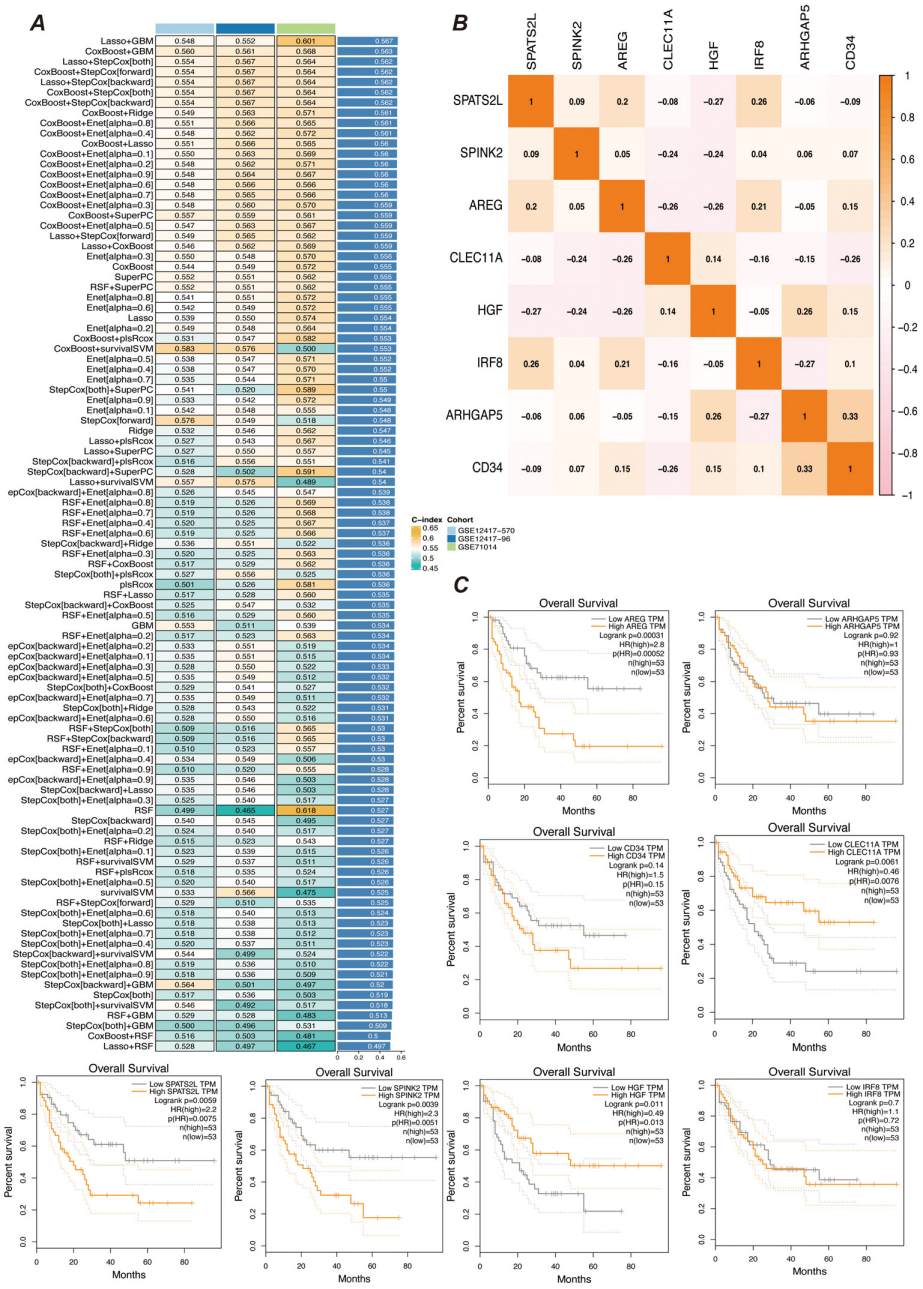
Prognostic gene selection. **(A)** Consensus model construction diagram. An illustration of the consensus model built using 101 different algorithm combinations, showing the different algorithm combinations and their consistency assessment results during the model construction process. **(B)** Heatmap of prognostic gene correlations. It displays the correlations between the selected prognostic genes. The color intensity in the heatmap indicates the strength of gene correlations, helping to identify key prognostic genes and their interrelationships. **(C)** K-M survival curves for gene expression high and low groups. Kaplan-Meier survival curves are plotted after grouping samples into high and low expression groups based on gene expression levels. The curves show differences in survival rates between gene expression level groups to assess their role in prognosis.

Abnormal proliferation and dysregulated differentiation of myeloid precursor cells contribute to the onset and progression of AML, and we wish to screen genes with prognostic value based on myeloid precursor cells for AML patients (26, 27). We used the marker genes of myeloid precursor cells from single-cell data and modeled them by a combination of ten machine learning algorithms, and the results showed that the combination of Lasso and GBM was the most effective (**Figure 4A**). The Lasso and GBM algorithms resulted in eight signature genes, SPATS2L, SPINK2, AREG, CLEC11A, HGF, IRF8, ARHGAP5, CD34, and their correlation was demonstrated by heatmap (**Figure 4B**). We plotted the K-M curves for each characterized gene using the corresponding overall survival data (**Figure 4C**).

### 3.5 Construction of prognostic models

**Figure 5:**
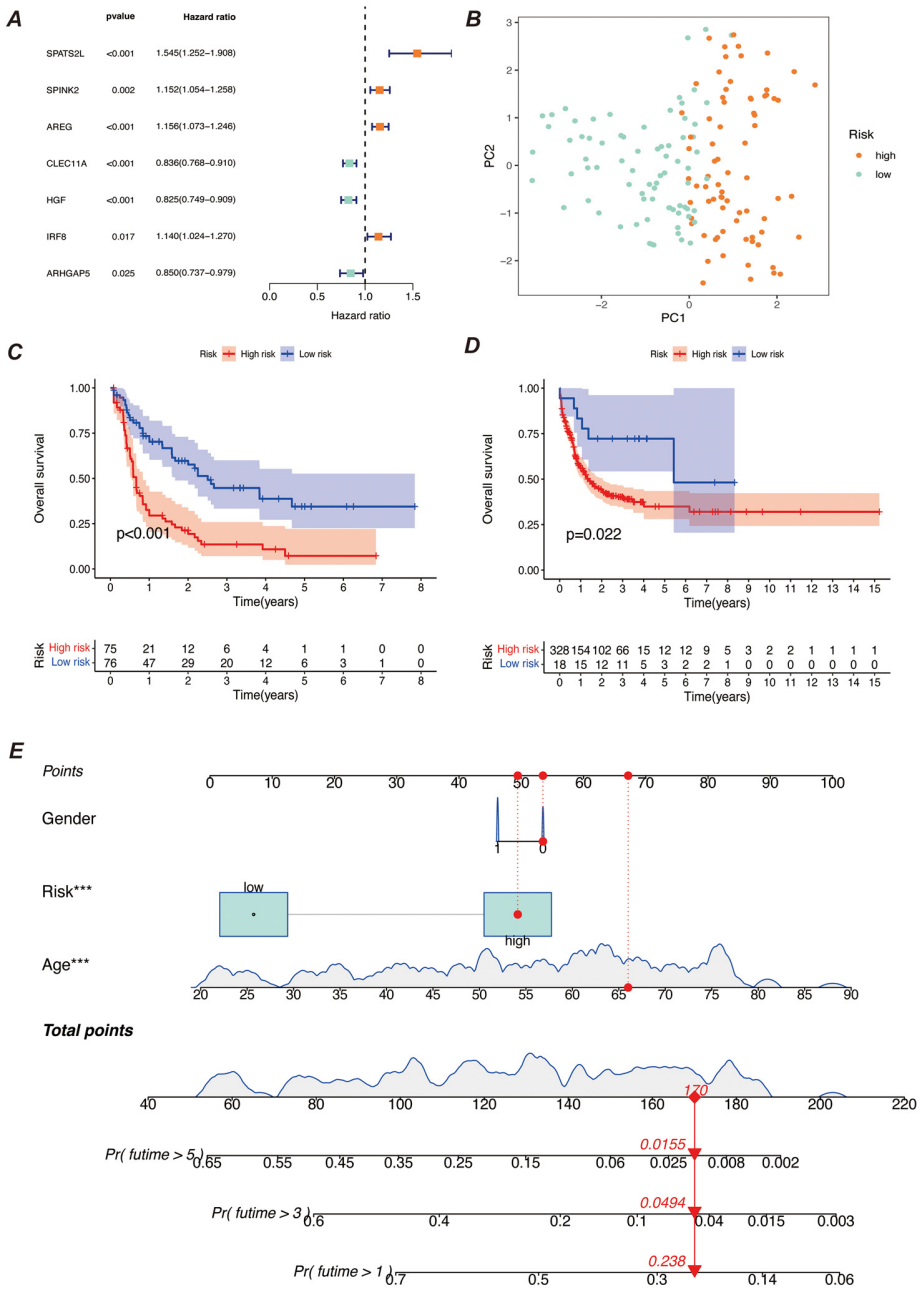
Construction of the prognostic model. **(A)** Meta-analysis diagram of univariate Cox survival analysis. It shows the results of meta-analysis of univariate Cox regression analysis for different prognostic factors to assess the correlation of each factor with survival time. The diagram includes the HR and 95% confidence interval for each factor. **(B)** PCA diagram of high-risk and low-risk groups. It compares the data distribution after dividing samples into high-risk and low-risk groups based on risk scores. The PCA diagram shows the distribution difference of the two groups on the principal components. **(C, D)** K-M survival curves. **(C)** shows the survival curves of high-risk and low-risk groups in the training set; **(D)** shows the survival curves of the corresponding groups in the validation set. Both diagrams are used to compare survival differences between different risk groups and to assess the prognostic predictive ability of the model. **(E)** Bar chart of comprehensive risk scores and clinical characteristics. It shows the association between comprehensive risk scores and clinical features, with the bar chart indicating the weight of each clinical characteristic in the comprehensive risk score and its predictive value for prognosis. *** indicates that the factor is statistically significant for disease prognosis with a p-value of less than 0.001.

**Figure 6:**
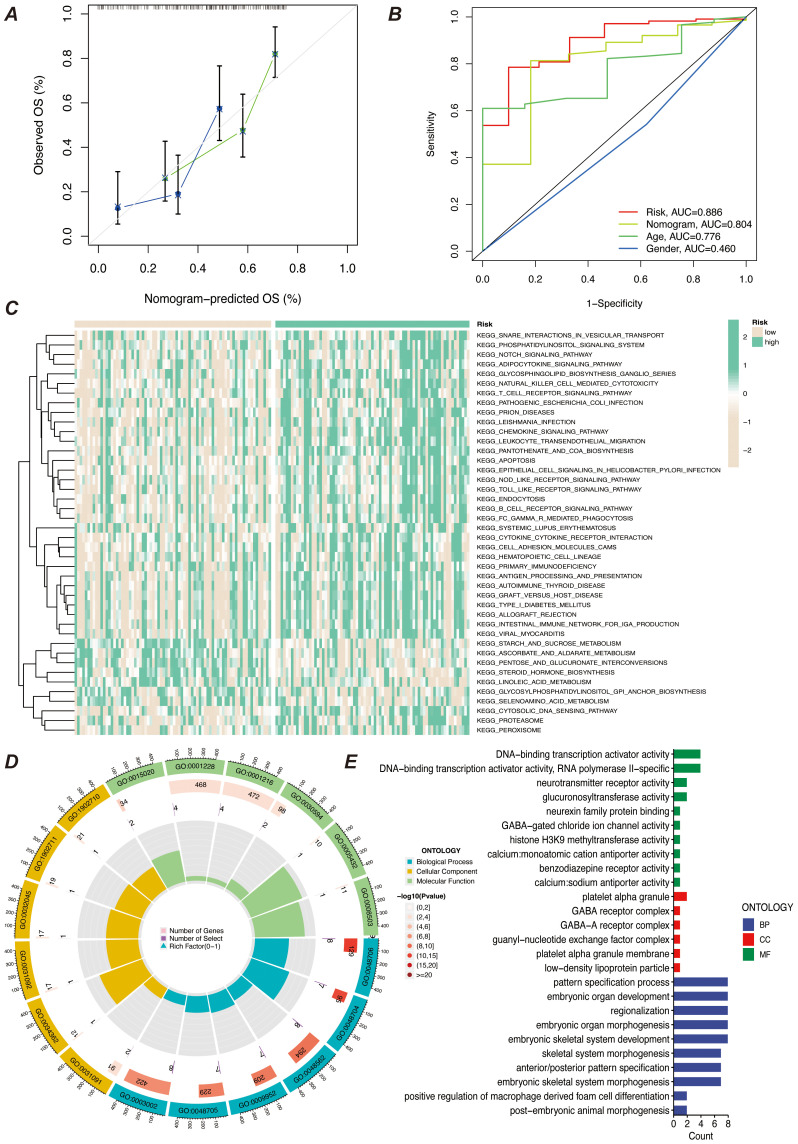
Predictive efficacy and functional enrichment analysis of the prognostic model. **(A)** Calibration curve of the prognostic model’s predictive efficacy. It shows the match between the model’s predicted risk scores and actual observed results, assessing the model’s predictive accuracy. **(B)** ROC curve of the prognostic model’s efficacy. It displays the receiver operating characteristic (ROC) curve of the model and the AUC, used to assess the model’s classification ability. **(C)** KEGG pathway enrichment analysis heatmap. It shows the enrichment of prognostic genes in KEGG pathways, helping to identify key biological pathways. **(D)** GO enrichment analysis bubble chart. It shows the enrichment of prognostic genes in GO categories, with the bubble chart displaying the relationships and enrichment levels of different GO terms. **(E)** GO enrichment analysis bar chart. It shows the enrichment levels of prognostic genes in different GO categories, with the bar chart reflecting the significance of each GO category.

We screened the characterized genes with p-value less than 0.05 as myeloid precursor cell-associated prognostic genes, which were SPATS2L, SPINK2, AREG,CLEC11A, HGF, IRF8, ARHGAP5. we demonstrated the p-value and HR value of each gene by forest plot (**Figure 5A**). We categorized patients into high and low risk groups based on the expression of myeloid precursor cells (**Figure 5B**). The prognostic model demonstrated good discrimination and prognostic value in both the training group data and the validation group data (**Figures 5C, D**). We constructed prognostic column-line plots by combining the risk score with age and gender, thereby predicting the likelihood of patient survival at 1, 3, and 5 years from the composite score (**Figure 5E**). The calibration curves demonstrated the accuracy of the model in predicting the likelihood of survival at 1, 3, and 5 years (**Figure 6A**).The AUC scores of the ROC demonstrated the reliability of our risk model and the column-line diagrams (**Figure 6B**).

### 3.6 High and low risk group enrichment analysis

To explore the characteristics of patients in the high- and low-risk groups under the myeloid precursor cell prognostic model, we performed KEGG and GO enrichment analyses. the KEGG results showed that immune-related pathways were less enriched in the high-risk group, while metabolic and signaling pathways were higher (**Figure 6C**). For example, Natural Killer Cell Mediated Cytotoxicity, the pathway showed low enrichment in the high-risk group. Natural killer cells are an important part of the innate immune system, responsible for recognizing and destroying cancerous or virally infected cells. reduced NK cell activity may imply that immune surveillance is impaired in the high-risk group, making it easier for leukemia cells to evade clearance by the immune system, further exacerbating the disease (28–30). The T Cell Receptor Signaling Pathway, a pathway that is also higher in the high-risk group, was also found to be less active in the high-risk group (**Figure 6C**). T cells are at the core of the adaptive immune response, recognizing antigens and activating the immune response through the T cell receptor (TCR), and the low enrichment of the T cell signaling pathway suggests that patients in the high-risk group may be immunosuppressed and unable to effectively initiate an immune response against leukemia cells, which may be related to the immune escape mechanism of the leukemia cells (31). The results of the enrichment of GO function showed that patients in the high-risk group had impaired immune surveillance function, making it easier for leukemia cells to evade the immune system and further exacerbate the disease. results showed that the high-risk group was enriched for more functions related to transcriptional regulation and metabolism, while the low-risk group exhibited more functions related to development and differentiation (**Figures 6D, E**). From the results of these enrichment analyses, there were significant differences in immune system function, cellular differentiation, and metabolic regulation between patients in the AML high- and low-risk groups. Patients in the high-risk group exhibited suppressed immune function, activation of metabolic pathways (e.g., GPI-anchored synthesis, proteasome pathway), and abnormalities in gene transcriptional activation (e.g., increased activity of transcription factors, increased activity of histone methylation), which may contribute to the proliferation and survival of leukemic cells. In contrast, patients in the low-risk group were more enriched for functions related to cell differentiation, immune regulation, and developmental processes, suggesting that these patients may have more normal hematopoietic functions and immune responses.

### 3.7 Immune infiltration analysis

**Figure 7:**
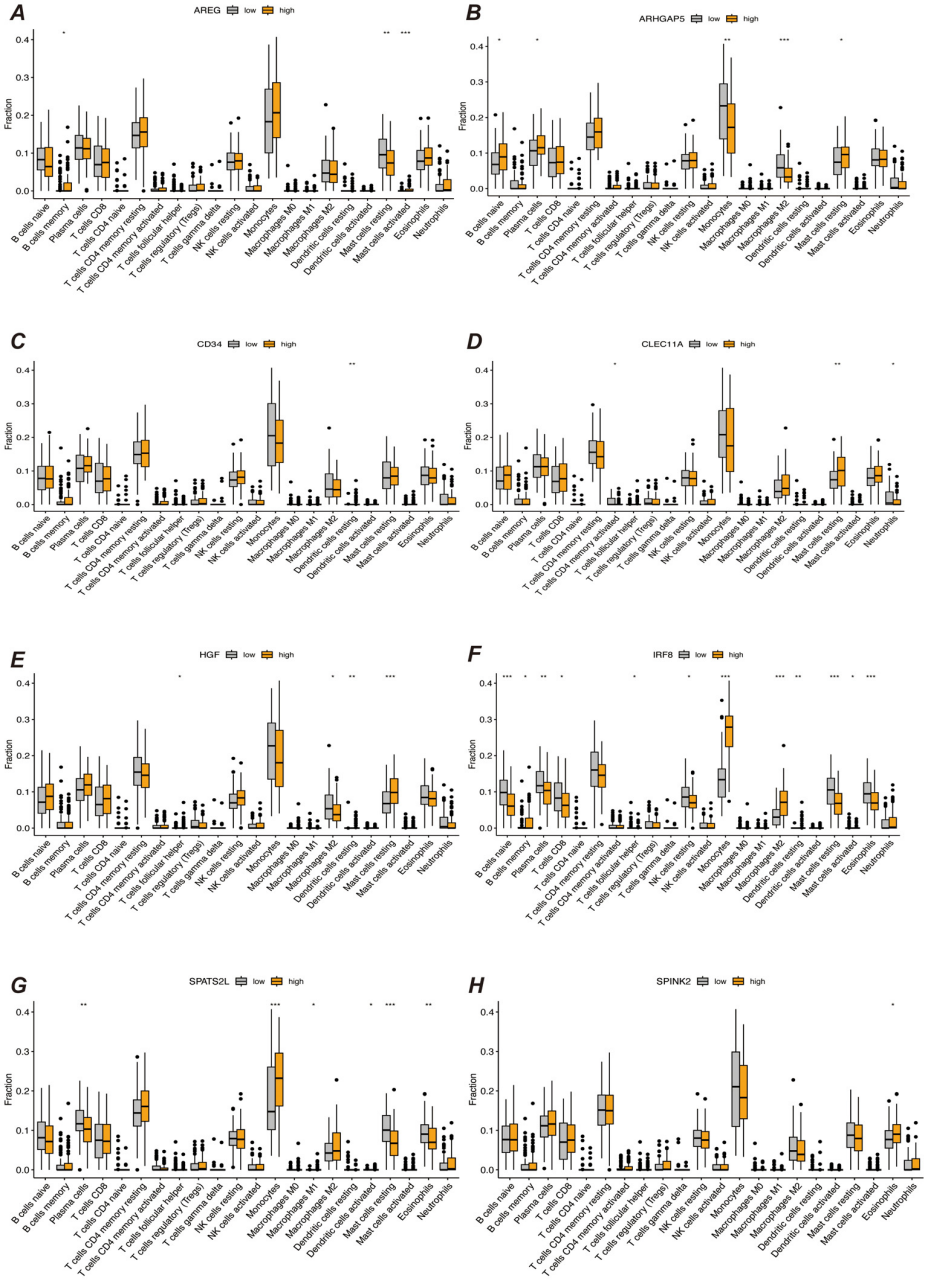
Immune infiltration analysis. **(A–H)** Results of immune infiltration analysis based on the expression levels of prognostic marker genes, grouped into different groups. Each subfigure shows the immune cell infiltration between different groups, comparing the immune infiltration levels between high and low expression groups of prognostic marker genes. “*” represents a p-value less than 0.05, “**” represent a p-value less than 0.01, and “***” represent a p-value less than 0.001.

**Figure 8:**
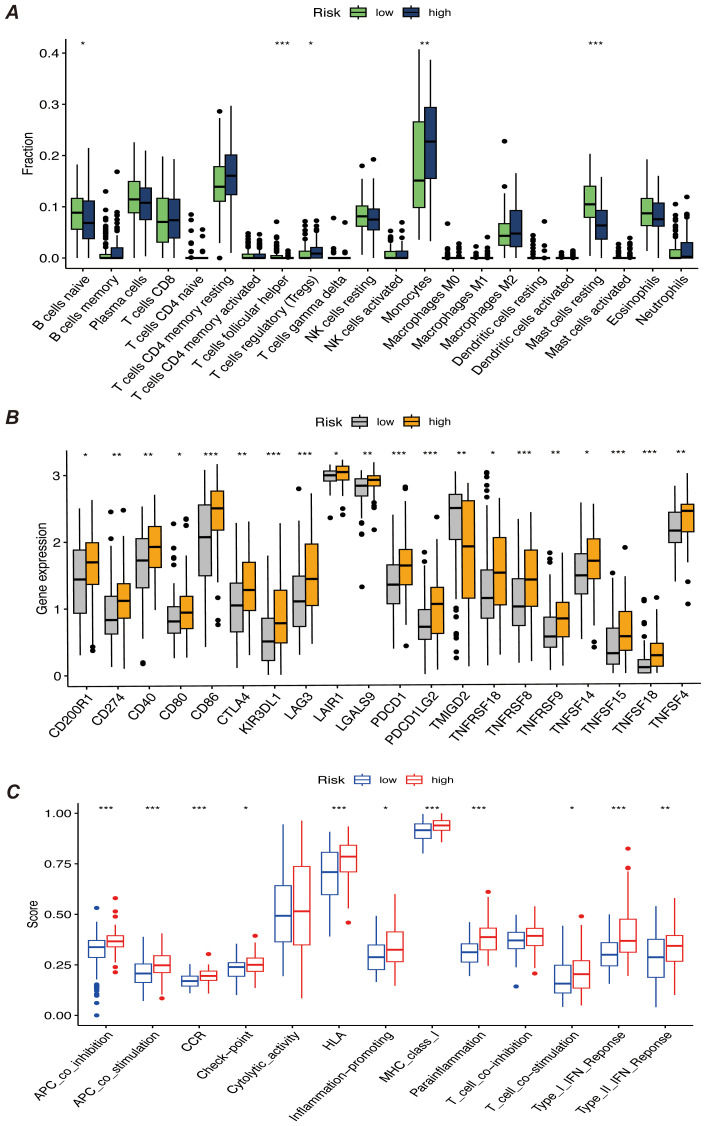
**(A)** Bar chart of immune infiltration analysis results for high-risk and low-risk patients, showing the infiltration levels of immune cells in different risk groups. **(B)** Bar chart comparing the expression levels of immune checkpoint genes, showing differences in the expression of major immune checkpoint genes between high-risk and low-risk groups. **(C)** Bar chart of immune function score results, comparing the immune function scores of high-risk and low-risk patients. “*” represents a p-value less than 0.05, “**” represent a p-value less than 0.01, and “***” represent a p-value less than 0.001, indicating the statistical significance of differences between groups.

We first performed immune infiltration analyses on subgroups of patients with the eight myeloid precursor cell signature genes, thus observing the consistency and differences in their effects on the tumor microenvironment (**Figures 7A–H**). Immune infiltration analyses were also performed on the high- and low-risk groups, and the high-risk group had higher levels of monocyte and macrophage M0-type infiltration. This may imply that macrophage polarization in the tumor microenvironment is associated with the progression of AML, and that M0-type macrophages are in an unpolarized state, where they may not yet have fully exerted their antitumor effects in the tumor microenvironment, and may even contribute to the growth of leukemic cells. The low-risk group showed a higher proportion of activated natural killer cells and activated mast cells, which may suggest that anti-tumor immune surveillance mechanisms are still more active in low-risk patients. Regulatory T cells were in higher proportion in the high-risk group. By suppressing the immune response, regulatory T cells may help leukemia cells to evade the attack of the immune system and further promote disease progression. The results of immune checkpoint analysis showed that the expression of classical immune checkpoint molecules, such as PDCD1, CD274, and CTLA4, was significantly elevated in the high-risk group. This suggests that leukemia cells in high-risk patients may inhibit the activity of the immune system through these checkpoint molecules, allowing tumor immune escape and thus promoting disease progression. The expression of the novel immune checkpoints, such as LAG3, TIM3, and TIGIT, was likewise higher in the high-risk group, suggesting that these molecules may play a key role in immunosuppression in high-risk AML patients. LAG3 and TIM3, in particular, have been recognized as potential therapeutic targets in recent years and may be closely related to immune escape mechanisms. The lower expression levels of these immune checkpoints in the low-risk group may reflect the relatively more active immune system of these patients, which is capable of recognizing and clearing leukemia cells more effectively. The results of the immune function score analysis showed that APC co-inhibition and Check-point scored higher in the high-risk group, suggesting that the activity of antigen-presenting cells was suppressed in high-risk patients and that the immune system may have difficulty in effectively initiating an anti-leukemia response. In addition, increased immune checkpoint activity implies activation of immunosuppressive pathways, further suppressing T-cell function. Inflammation-promoting scored higher in the high-risk group, which may be related to the chronic inflammatory state in the tumor microenvironment. Chronic inflammation may promote cancer cell survival and proliferation in some cases. Type I IFN response and Type II IFN response scored higher in the low-risk group, suggesting that low-risk patients may have stronger antiviral and antitumor immune responses. Together, these analyses reveal that there are significant differences in the immune environments of high- and low-risk AML patients, that immunosuppression is an important mechanism of disease progression in the high-risk group, and that immunotherapies (e.g., checkpoint inhibitors) may have a positive therapeutic effect in these patients (**Figures 8A–C**).

### 3.8 Prognostic marker gene expression validation and enrichment analysis

**Figure 9:**
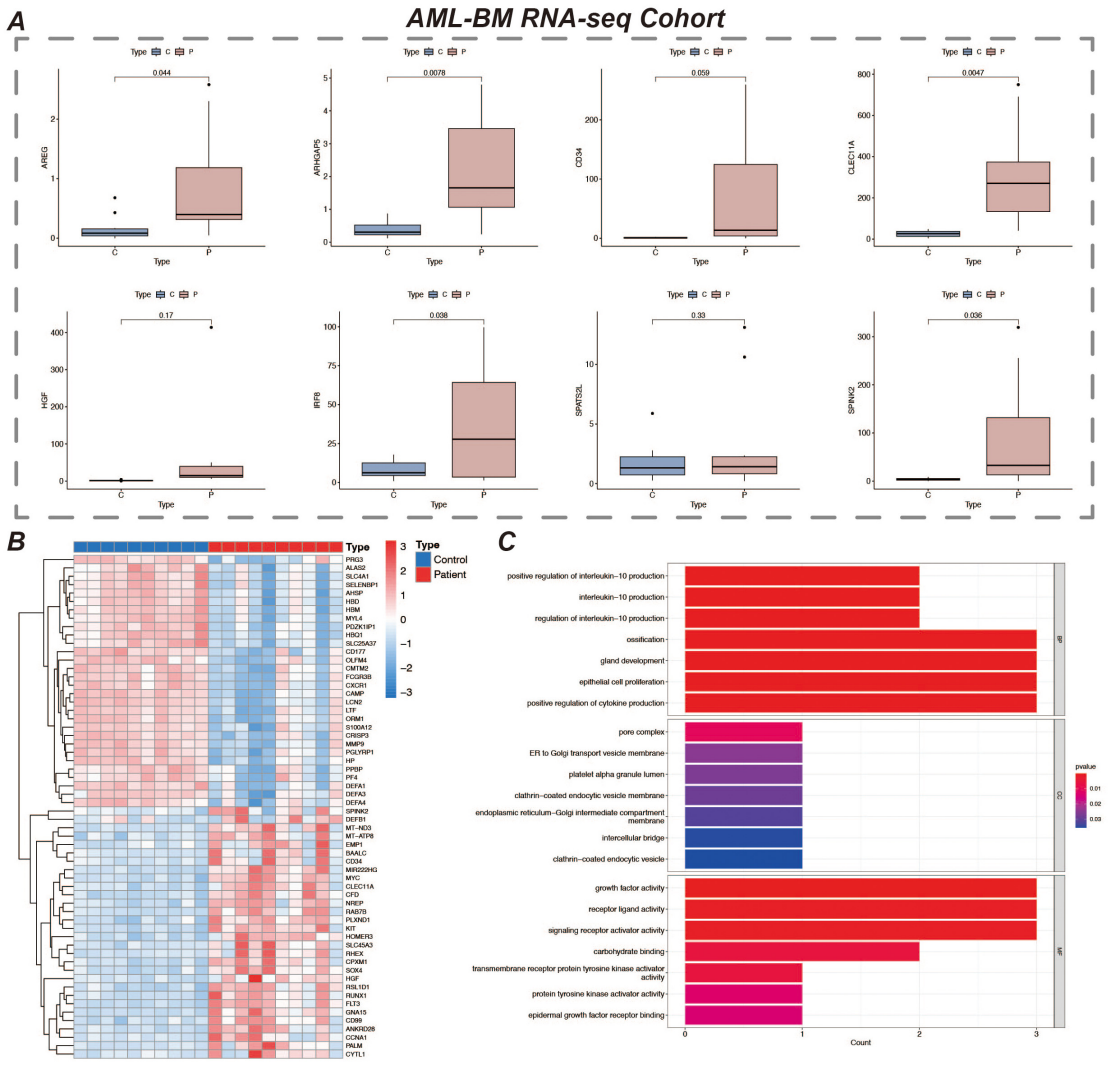
Expression of prognostic marker genes and GO enrichment analysis. **(A)** It shows the expression of 8 prognostic marker genes in the AML-BM RNA-seq Cohort. The figure displays the expression levels of these genes in samples, revealing their expression characteristics in AML bone marrow samples. **(B)** Heatmap of differentially expressed genes in the normal and disease groups, showing 30 genes each that are up- and down-regulated. Red represents up-regulated expression and blue represents down-regulated expression. **(C)** Bar chart of GO enrichment analysis results.

We validated the expression of prognostic marker genes using the AML-BM RNA-seq Cohort with the aim of confirming the differences in the expression of these genes between acute myeloid leukemia (AML) patients and normal controls. By comparing the data from the AML group with that of the normal group, we used an independent samples t-test to statistically analyze the gene expression. The results showed that seven prognostic marker genes showed significant expression differences between the AML and normal groups (**Figure 9A**). We performed gene differential expression analysis using normal and disease samples from the AML-BM RNA-seq cohort and demonstrated the top thirty and bottom thirty differentially expressed genes by heatmap (**Figure 9B**). Then based on the differentially expressed genes, we performed GO enrichment analysis and demonstrated the enriched active pathways by bar graph (**Figure 9C**).

## 4 Discussion

In this study, we comprehensively analyzed the tumor microenvironment and prognosis-related genes in acute myeloid leukemia (AML) and constructed a prognostic model for AML patients based on single-cell RNA sequencing (scRNA-seq) and RNA-seq data. Our study revealed the abnormal proliferation and dysregulated differentiation of myeloid precursor cells in AML, elucidating the important role of this cell type in the tumor microenvironment and its potential as a potential therapeutic target for AML. Through systematic cell communication analysis, mimetic timing analysis and enrichment analysis, we not only deeply explored the function and developmental trajectory of myeloid precursor cells, but also screened out genes that are closely related to patient prognosis.

Firstly, we successfully identified myeloid precursor cells and other major cell types through dimensionality reduction clustering and cell type identification of single-cell data. Myeloid precursor cells are significantly more prevalent in AML patients, a finding that suggests their critical role in the pathological process of AML. Abnormal proliferation of these cells may be closely related to the development of AML, especially their abnormal activity in the tumor microenvironment (32, 33). Mimetic time-series analysis further validated the developmental trajectory of myeloid precursor cells, showing their extensive proliferation in AML and peaking in the late stage of the disease. Combined with the results of enrichment analysis, the gene expression profile of myeloid precursor cells revealed their specific functions in AML, especially the activation of the MIF signaling pathway, which may be related to the proliferation and autocrine regulation of myeloid precursor cells (34, 35).

Cell communication analysis also revealed for us the complex interactions between myeloid precursor cells and other cell types in the AML tumor microenvironment. We found that myeloid precursor cells interacted with NK cells and monocytes particularly in the AML microenvironment. the role of NK cells was significantly reduced in the high-risk group, possibly suggesting that the immune system of these patients was significantly suppressed. This was further verified in our analysis of immune infiltration in the high- and low-risk groups, which demonstrated suppression of immune function with activation of immune escape mechanisms in the high-risk group (36). These findings emphasize the association between immune microenvironment characteristics and disease progression in AML patients and provide a theoretical basis for targeted immunotherapy (37).

Based on the marker gene screening of myeloid precursor cells, we constructed a prognostic model for AML and selected a combined model of Lasso and GBM by comparing multiple machine learning algorithms. The final 8 characterized genes (SPATS2L, SPINK2, AREG, CLEC11A, HGF, IRF8, ARHGAP5, CD34) showed significant prognostic value in patients’ survival analysis. These genes may not only help predict the prognosis of AML patients but may also serve as potential targets for future therapy (38–40). For example, high expression of SPINK2 and AREG is closely associated with malignant progression of AML, suggesting their role in disease regulation. By K-M curves and Cox regression analysis, we further validated the expression differences of these genes in patients of high and low risk groups, and the good performance of the prognostic model was further supported by the data from the training and validation groups (41).

To further validate the expression patterns of these marker genes and their actual roles in AML patients, we collected bone marrow samples from 10 AML patients and 10 healthy individuals and performed RNA-seq sequencing analysis. The results showed that some of the prognosis-related genes screened in this paper were significantly highly expressed in bone marrow samples from AML patients and were statistically significant compared with normal individuals (42). This result is consistent with our findings obtained from publicly available databases and bioinformatics analyses, further enhancing the reliability of these genes as potential prognostic markers for AML. In addition, the gene expression data revealed that certain genes may play key regulatory roles in the development of AML, providing direction for subsequent mechanistic studies (43).

In addition, our immunoassays revealed significant features of high-risk AML patients in terms of immune escape, especially the high expression of classical immune checkpoint molecules (e.g., PDCD1, CTLA4), which further suggests that these patients may escape from the attack of the immune system through immune checkpoint inhibition mechanisms (44–46). Patients in the high-risk group had higher levels of M0-type macrophage infiltration, whereas the low-risk group showed greater immune surveillance. This suggests that the role of the immune system in the progression of AML patients is crucial, and future treatment in combination with immune checkpoint inhibitors or other immunotherapies may be needed to improve the prognosis of patients in the high-risk group (47, 48).

In summary, this study constructed a prognostic model of AML by systematic single-cell analysis and machine learning modelling and revealed the critical role of myeloid precursor cells in the pathological process of AML. Future studies should further validate the prognostic value of these genes in independent cohorts and explore myeloid precursor cell-based therapeutic interventions, thus providing new ideas for individualized treatment of AML patients.

## 5 Conclusion

In this study, the cellular heterogeneity and potential molecular mechanisms in the tumor microenvironment of acute myeloid leukemia (AML) were deeply resolved by integrating single-scRNA-seq and bulk RNA-seq data. The aberrant proliferation of myeloid precursor cells and their critical role in AML development were revealed by cellular communication, mimetic time-series analysis, and screening of prognosis-related genes. The AML prognostic model constructed based on survival analysis identified a variety of prognostic-related genes and demonstrated their potential application value in survival prediction of AML patients. In addition, this study revealed features related to immunosuppression and tumor immune escape, which provided new ideas and potential targets for personalized treatment of AML.

## Data Availability

The data presented in the study are deposited in the Dryad repository, accession link: https://doi.org/10.5061/dryad.h9w0vt4t2.
